# Impact of serum sodium trajectory on 30-day mortality in traumatic brain injury patients: insights from a retrospective cohort study using MIMIC-IV database

**DOI:** 10.3389/fneur.2025.1618586

**Published:** 2025-07-25

**Authors:** Shuangshuang Huang, Songmin Huang, Wulei Wang, Zhimin Li, Yingjun Liu

**Affiliations:** ^1^Ningbo Haishu Traditional Chinese Medicine Hospital, Ningbo, Zhejiang, China; ^2^Xiangshan County Traditional Chinese Medicine Hospital, Xiangshan, Zhejiang, China; ^3^The Third Affiliated Hospital of Zhejiang Chinese Medical University (Zhongshan Hospital of Zhejiang Province), Hangzhou, Zhejiang, China

**Keywords:** traumatic brain injury, sodium, trajectory, latent growth mixture model, MIMIC-IV database

## Abstract

**Background:**

Hypernatremia is frequently encountered in individuals with traumatic brain injury (TBI), and research has demonstrated a correlation between serum sodium levels and patient outcomes in TBI cases. This study aims to explore the temporal patterns of serum sodium concentrations and assesses their prognostic significance in TBI patients.

**Methods:**

This study employed data sourced from the database of Medical Information Mart for Intensive Care IV (MIMIC-IV). We applied a latent growth mixture model (LGMM) to construct the serum sodium trajectories of TBI patients within the first 96 h of their intensive care unit (ICU) stay, based on mean serum sodium measurements taken at 24-h intervals. Subsequently, Cox regression models were employed to analyze the associations among initial serum sodium levels, serum sodium trajectories, and mortality outcomes of 30 and 90 day.

**Results:**

A total of 852 TBI patients were included, and the LGMM model categorized serum sodium trajectories into 4 classes. Significant differences in prognosis were observed between the different grades of TBI patients, with the worst prognosis for patients with TBI in Class 2 (slow-growth type) compared to Class 1 (normal stable type) and no significant difference in mortality for the remaining grades. In addition, after adjusting for confounding factors, high first serum sodium levels were related to higher 30-day (HR = 2.14, 95% CI: 1.13–4.04, *p* = 0.019) and 90-day (HR = 2.22, 95% CI: 1.21–4.08, *p* = 0.01) mortality rates in TBI patients.

**Conclusion:**

Both first-time serum sodium and serum sodium trajectory were independent influences on the prognosis of TBI patients. Thus, clinicians should closely monitor serum sodium in TBI patients and adjust treatment strategies based on its dynamic changes.

## Introduction

Traumatic brain injury (TBI) is an acquired brain injury caused by external mechanical forces (1). Among all common neurological disorders, TBI has the highest incidence rate (2), and about 69 million people suffer from TBI every year (3), causing a heavy social burden. TBI is not only an acute condition but also leads to long-term complications, making it a leading cause of global morbidity and mortality (2, 4, 5).

Hypernatremia is the most common electrolyte imbalance observed in patients with TBI (6). Hypernatremia can be caused by various factors, with central urosepsis being the most frequent, along with the administration of hypertonic fluids and sodium-based medications, insufficient rehydration, or excessive water loss (7). Many studies have emphasized that hypernatremia is associated with a higher risk of death and acts as an independent prognostic indicator for patients with severe TBI (8, 9). We conducted a retrospective analysis on TBI patients, categorizing them according to the peak serum sodium levels recorded within the first 24 h of admission. Their researches showed an L-shaped link between sodium concentrations and mortality, noting a marked increase in mortality when serum sodium exceeded 144.1 mmol/L (10). However, the serum sodium level of TBI patients during hospitalization changes dynamically and assessing the mortality and prognosis of TBI patients based only on the maximum value of serum sodium at the time of admission might be incomplete and inaccurate.

The trajectory of serum sodium change offered a more comprehensive reflection of longitudinal variations in serum sodium levels than traditional variability indicators. However, the link between changes in serum sodium levels and outcomes in TBI patients is still not well understood. In this investigation, we identified the trends of serum sodium in TBI patients during the initial 96 h of intensive care unit (ICU) stay and assessed their relationship with mortality rates at 30 and 90 days. Our findings aim to inform clinical strategies for serum sodium management in TBI patients.

## Materials and methods

### Data source

The research utilized data from the Medical Information Mart for Intensive Care IV (MIMIC-IV), a large-scale public repository containing de-identified clinical records of ICU patients at Beth Israel Deaconess Medical Center between 2008 and 2019 (11). This database includes a wide range of data such as demographics, laboratory tests, vital signs, medication records, nursing notes, and prognostic information. By successfully completing the National Institutes of Health’s online training and certification exam, Shuangshuang Huang obtained access to the MIMIC-IV database (Record ID: 66948192). Since MIMIC-IV database was a publicly available de-identified database and the study was retrospective, the Institutional Review Board of the Ningbo Haishu Traditional Chinese Medicine Hospital waived the requirement for informed consent.

### Study population

Patient data with TBI, identified through ICD-9 and ICD-10 codes, was collected for the study, as noted in Supplementary material. The exclusion criteria were: (1) age less than 18 years; (2) patients who stayed in the ICU for under 96 h; (3) patients who did not measure serum sodium every 24 h. Only data from the initial ICU admission were considered for patients with more than one ICU stay. Since the study utilized de-identified patient data, individual informed consent was waived.

### Exposure

In our research, we examined the average variations in serum sodium levels over the initial four 24-h periods after ICU admission as exposure factors. Based on the initial serum sodium level, we categorized into a hypernatremia group (>145 mmol/L) and a moderate hyponatremia group (≤145 mmol/L). Simultaneously, a latent growth mixture model (LGMM) was applied to map out the changes in serum sodium levels during the initial 96 h following ICU admission. Specifically, given that serum sodium levels were inherently dynamic and fluctuate depending on factors such as individual clinical progress or therapeutic interventions. This resulted in significant heterogeneity in sodium level trajectories between patients. The LGMM is particularly well suited to capture this complexity because, unlike traditional methods, the LGMM categorizes individuals into potential categories with similar trajectory patterns, while accounting for variability within each category. At the same time, it provides a more flexible and realistic representation of serum sodium dynamics by incorporating random effects that allow for variation between individuals within the same trajectory category. The LGMM was selected due to its superior adaptability to the nuanced, patient-specific evolution of sodium levels compared to population-based trajectory modeling or standard clustering methods (12). The best models usually need to fulfill the following conditions: ① A better model fit is indicated by lower values for both the Akaike Information Criterion (AIC) and the Bayesian Information Criterion (BIC); ② The entropy should be at least 0.7, and a higher log-likelihood value is preferable; ③ The average posterior probability should exceed 0.7, indicating a high degree of consistency between the members and their respective trajectories; and ④ The proportion of each category should be at least 1% (13, 14). In determining the optimal number of trajectory categories, we considered a number of factors. The 4-class model demonstrated a better model fit compared to models with fewer categories, as evidenced by the smaller AIC and BIC and entropy values greater than 0.7, which represented a clearer categorization. In terms of clinical interpretability, the slow-growth trajectory (Class 2) represented a clinically significant high-risk subgroup (20.31% of the cohort). Although the Class 5 model showed a slightly better AIC, the proportion of high-risk patients (18.08%) did not show a substantial difference. In line with the principle of model parsimony, the Class 4 model achieved an optimal balance between complexity and interpretability by avoiding excessive segmentation while maintaining clinical relevance. Consequently, the present study categorized the blood sodium trajectories into 4 classes. The log likelihood, AIC, BIC, entropy values, and percentages of the models corresponding to the different categories are shown in Supplementary Table 1.

### Outcome

This study primarily aimed to evaluate the 30-day all-cause mortality after ICU admission in TBI patients, while the 90-day mortality served as a secondary measure.

### Data collection

The data collected in this study consisted of seven categories, specifically demographics, vital signs, disease severity scores, comorbidities, medication usage, laboratory indicators of first ICU admission, and other variables. The specifics of each category are presented in Table 1.

**Table 1:** Collected variables.

Items	Content
Demographics	Gender, age, race
Vital signs	Temperature, heart rate, respiratory rate (RR), systolic blood pressure (SBP), diastolic blood pressure (DBP), and oxyhemoglobin saturation (SpO2)
Disease severity scores	Sequential Organ Failure Assessment (SOFA), Acute Physiology Score III (APSIII), Glasgow Coma Scale (GCS), Charlson Comorbidity Index (CCI), Simplified Acute Physiology Score II (SAPSII), Oxford Acute Severity of Illness Score (OASIS)
Comorbidities	Hypertension, diabetes mellitus, myocardial infarction, congestive heart failure, chronic obstructive pulmonary disease (COPD), chronic kidney disease (CKD)
Medication usage	Vasopressor and diuretic use
Laboratory indicators of first ICU admission	Hemoglobin, white blood cell count (WBC), blood urea nitrogen (BUN), prothrombin time (PT), partial thromboplastin time (PTT), platelet count, serum creatinine, calcium, glucose, serum sodium, potassium
Other variables	Ventilation use

### Statistical analysis

Frequencies and percentages [*n* (%)] were used to present categorical variables, whereas continuous data were shown as either mean ± standard deviation or median with interquartile range [M (Q1, Q3)]. Chi-square or Fisher’s exact tests were utilized for categorical variables, and the Kruskal-Wallis test was employed for continuous variables to compare the groups.

The associations between the initial serum sodium level upon ICU admission (a categorical variable) and the subsequent serum sodium trajectory within the first 96 h of ICU stay, as well as their impact on mortality risks, were investigated in our study. And then, the Hazard ratios (HRs) and 95% confidence intervals (CIs) for 30-day and 90-day mortality were calculated. Additionally, mortality risks across different serum sodium patterns were compared using Kaplan–Meier survival analysis and log-rank tests. Univariate Cox regression (model 1) was used to screen covariates linked to 30-day mortality (Supplementary Table 2), and those with a *p*-value less than 0.05 were included in further multivariate models for adjustment. In model 2, adjustments were made for age and sex, and model 3 added further adjustments for GCS, APSIII, OASIS, SOFA, SAPSII, CCI, and model 4 additionally included heart failure, diabetes mellitus, CKD, vasopressor use, temperature, calcium, serum creatinine, hemoglobin, RBC, and BUN. In addition, age, sex, CKD, diabetes, heart failure, and vasopressor use were analyzed in subgroups. Additionally, to verify the robustness of the findings, we performed two sensitivity analyses. We first repeated the main analyses after imputing missing values using mean interpolation, given that the proportion of missing data for all covariates was less than 5% (Supplementary Table 3). We then reanalyzed the remaining classes after excluding the Class 3 subgroup, which had a small sample size that may have affected the statistical validity, from the analysis of 30-day and 90-day mortality. Data processing and analysis in this study were performed using DecisionLinnc1.0 software (15) and R (version 4.4.2). The threshold for statistical significance was set at a two-tailed *p*-value of 0.05.

## Results

### Baseline characteristics

**Figure 1:**
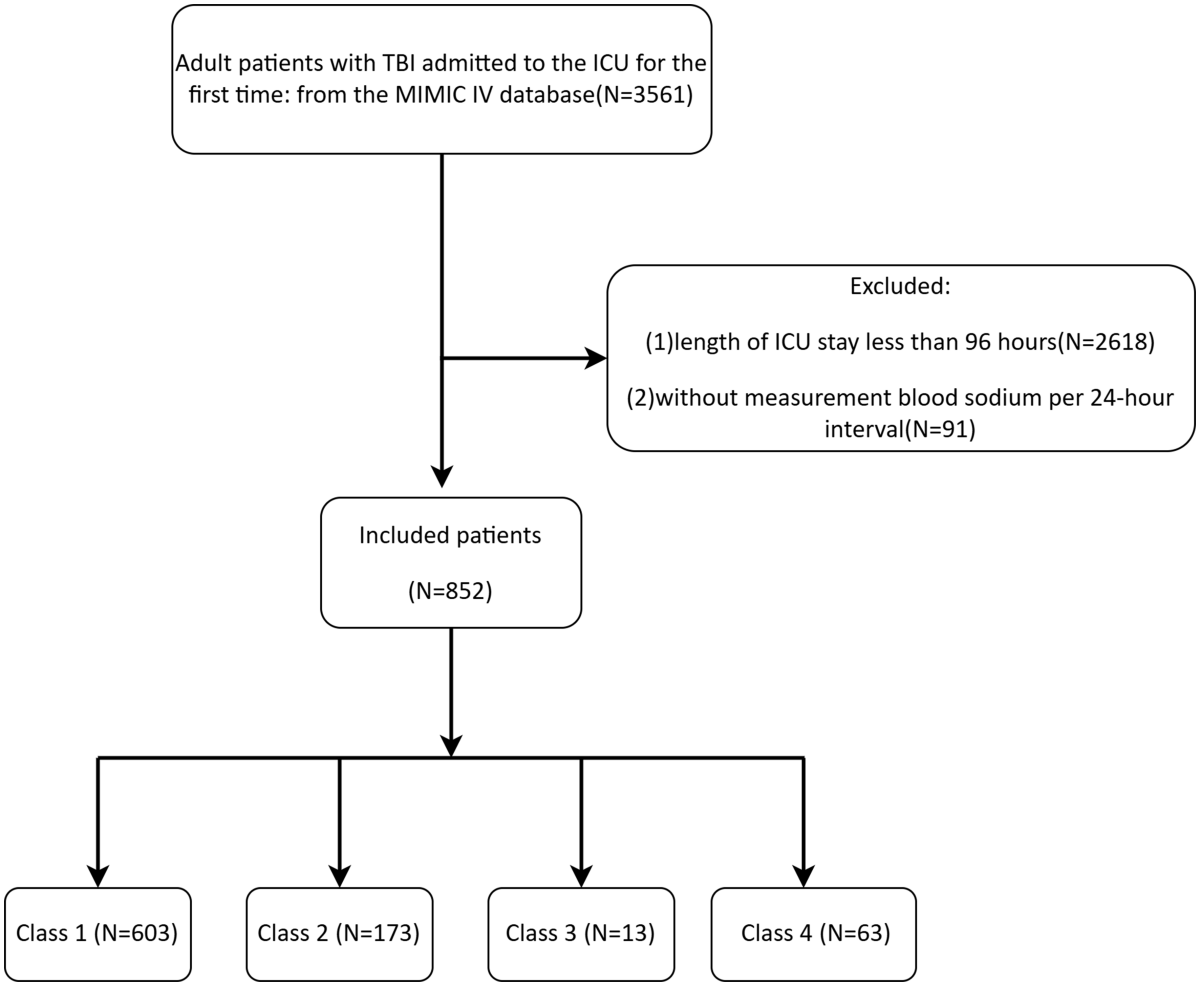
Flowchart for inclusion exclusion of TBI patients.

The MIMIC-IV database provided data on 852 TBI patients, with the detailed screening process shown in Figure 1. Table 2 showed the baseline characteristics of patients with TBI (including only clinically or statistically significant variables) divided on the basis of 4 categories of serum sodium level classes. Complete baseline characteristics data were detailed in Supplementary Table 4. Patients had a median age of 64 years, and most were male, accounting for 67.72%. There were 356 patients with comorbid hypertension (41.78%), 73 patients with CKD (8.57%), and 176 patients with diabetes mellitus (20.66%) with a more balanced distribution of gender and ethnicity in each group.

**Table 2:** Selected baseline characteristics of TBI patients by 4 serum sodium classes.

Variable	Overall	Class 1	Class 2	Class 3	Class 4	*p*
*N*	852	603	173	13	63	
Age	64.00 (44.00–78.00)	64.00 (46.00–79.00)	60.00 (35.00–74.00)	52.00 (30.00–64.00)	71.00 (56.00–81.00)	0.007
Gender (%)						0.564
Female	275 (32.28)	188 (31.18)	62 (35.84)	3 (23.08)	22 (34.92)	
Male	577 (67.72)	415 (68.82)	111 (64.16)	10 (76.92)	41 (65.08)	
Race (%)						0.548
White	460 (53.99)	335 (55.56)	83 (47.98)	6 (46.15)	36 (57.14)	
Black	47 (5.52)	34 (5.64)	8 (4.62)	1 (7.69)	4 (6.35)	
Others	345 (40.49)	234 (38.81)	82 (47.40)	6 (46.15)	23 (36.51)	
GCS	15.00 (12.00–15.00)	15.00 (12.00–15.00)	15.00 (12.00–15.00)	15.00 (15.00–15.00)	14.00 (12.00–15.00)	0.022
SpO2, %	100 (97–100)	100 (97–100)	100 (98–100)	100 (97–100)	99 (96–100)	0.002
Glucose, mg/dl	137.57 ± 37.10	136.45 ± 36.53	144.28 ± 38.43	131.05 ± 35.69	131.23 ± 37.44	0.029
WBC, k/ul	11.67 ± 4.44	11.47 ± 4.32	12.75 ± 4.51	10.75 ± 3.73	10.87 ± 5.06	0.001
Diuretic use (%)						<0.001
No	574 (67.37)	428 (70.98)	92 (53.18)	7 (53.85)	47 (74.60)	
Yes	278 (32.63)	175 (29.02)	81 (46.82)	6 (46.15)	16 (25.40)	
Hypertension (%)						0.743
No	496 (58.22)	354 (58.71)	96 (55.49)	9 (69.23)	37 (58.73)	
Yes	356 (41.78)	249 (41.29)	77 (44.51)	4 (30.77)	26 (41.27)	
CKD (%)						0.049
No	779 (91.43)	557 (92.37)	159 (91.91)	11 (84.62)	52 (82.54)	
Yes	73 (8.57)	46 (7.63)	14 (8.09)	2 (15.38)	11 (17.46)	
Diabetes (%)						0.887
No	676 (79.34)	476 (78.94)	140 (80.92)	11 (84.62)	49 (77.78)	
Yes	176 (20.66)	127 (21.06)	33 (19.08)	2 (15.38)	14 (22.22)	
30-day mortality (%)						0.003
Alive	710 (83.33)	520 (86.24)	129 (74.57)	10 (76.92)	51 (80.95)	
Dead	142 (16.67)	83 (13.76)	44 (25.43)	3 (23.08)	12 (19.05)	
90-day mortality (%)						<0.001
Alive	702 (82.39)	518 (85.90)	124 (71.68)	10 (76.92)	50 (79.37)	
Dead	150 (17.61)	85 (14.10)	49 (28.32)	3 (23.08)	13 (20.63)	

**Figure 2:**
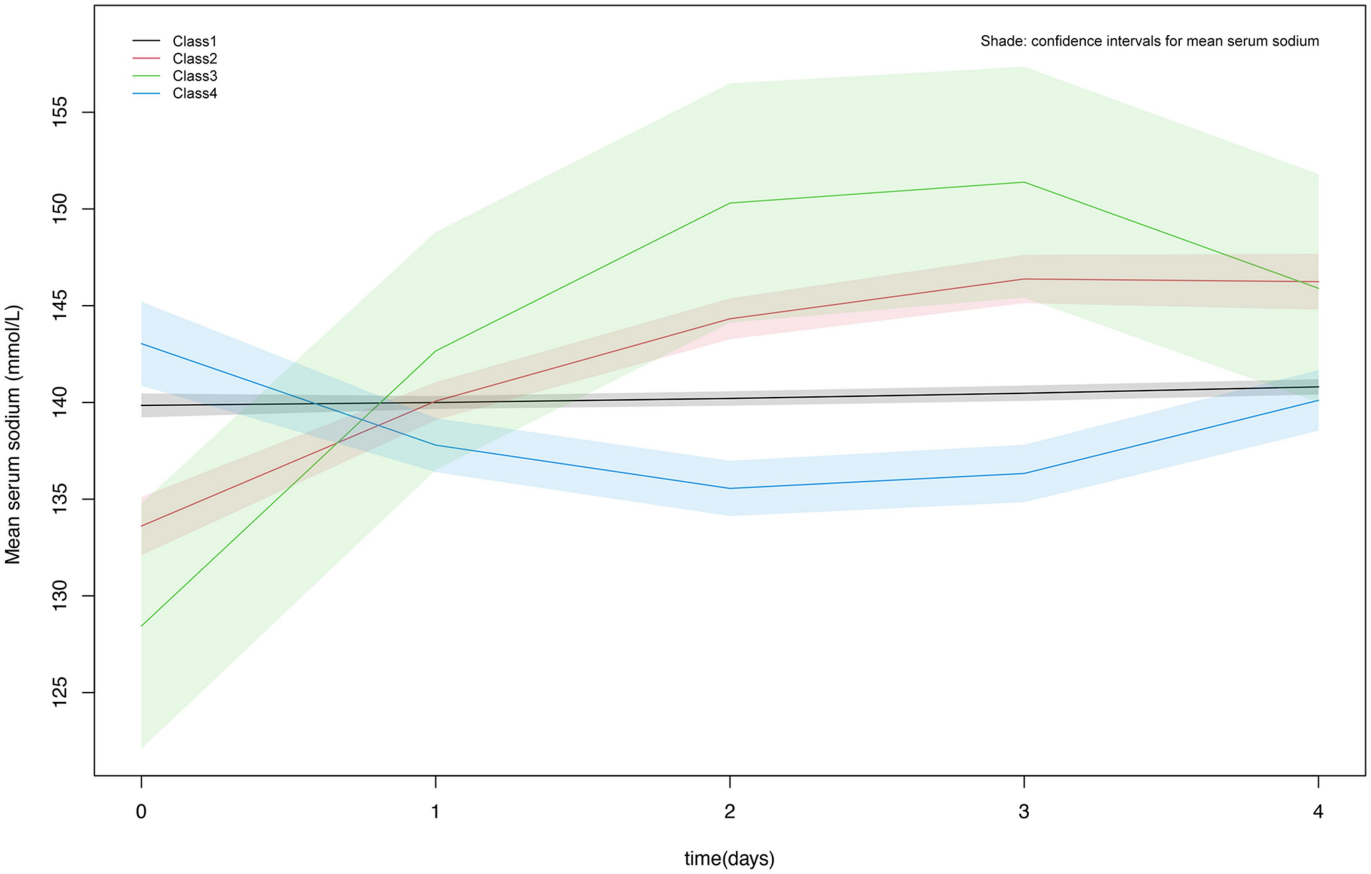
Classes of serum sodium levels in TBI patients at 96 h After ICU admission.

Figure 2 showed the detailed trajectory of serum sodium levels for the four classes: Class 1 was the normal stable type with the highest percentage (70.77%), totaling 603 individuals; Class 2 was slow-growth type with 173 individuals (20.31%); Class 3 exhibited a rapid increase followed by a decline with the lowest percentage of 13 individuals (1.53%); Class 4 had a slow decline followed by a gradual increase and it accounts for 7.39% of the total population, totaling 63 persons.

### Serum sodium classes and mortality

**Figure 3:**
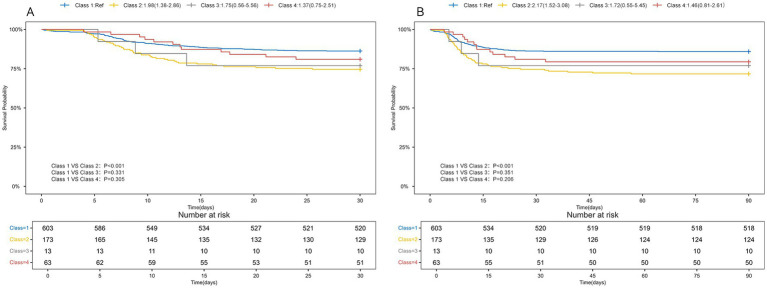
Kaplan-Meyer survival curves in TBI patients with different serum sodium classes. **(A)** 30-day Kaplan-Meier survival curves for TBI patients with different serum sodium levels; **(B)** 90-day Kaplan-Meier survival curves for TBI patients with different serum sodium levels.

Table 3 presented the associations between the initial serum sodium level (categorized) upon ICU admission and serum sodium categorization within 96 h of ICU admission with 30-day and 90-day mortality in TBI patients. Univariate Cox regression analysis showed no significant association between serum sodium levels (≤145 mmol/L or >145 mmol/L) and mortality at either time point. But after accounting for various confounding factors, serum sodium levels above 145 mmol/L were independently correlated with an increased risk of death within 30 days (HR = 2.14, 95% CI: 1.13–4.04, *p* = 0.019) and 90 days (HR = 2.22, 95% CI: 1.21–4.08, *p* = 0.010). In univariate Cox regression models, 30-day (HR = 1.98, 95% CI: 1.38–2.86, *p* < 0.001) and 90-day (HR = 2.17, 95% CI: 1.52–3.08, *p* < 0.001) mortality rates were higher in TBI patients in Class2 compared with Class1, with no statistical significance for the remaining classifications. In a multifactorial cox regression model, Class 2 was still associated with higher mortality. Kaplan–Meier survival curves indicated that Class 2 had the highest 30- and 90-day mortality rates, as shown in Figure 3.

**Table 3:** Correlation of serum sodium levels with 30-day and 90-day mortality among TBI patients.

Outcome	Model 1		Model 2		Model 3		Model 4	
	HR	*p*	HR	*p*	HR	*p*	HR	*p*
30-day mortality								
Serum sodium levels								
≤145 mmol/L	Ref		Ref		Ref		Ref	
>145 mmol/L	1.64 (0.95–2.85)	0.079	2.29 (1.31–4.01)	0.004	2.06 (1.15–3.69)	0.015	2.14 (1.13–4.04)	0.019
Serum sodium classes								
Class 1	Ref		Ref		Ref		Ref	
Class 2	1.98 (1.38–2.86)	<0.001	2.17 (1.50–3.14)	<0.001	2.06 (1.42–2.99)	<0.001	2.08 (1.42–3.05)	<0.001
Class 3	1.75 (0.56–5.56)	0.337	2.50 (0.79–7.93)	0.120	2.47 (0.76–7.97)	0.131	2.87 (0.88–9.42)	0.082
Class 4	1.37 (0.75–2.51)	0.309	1.20 (0.65–2.19)	0.571	1.02 (0.55–1.90)	0.941	1.07 (0.57–2.02)	0.825
90-day mortality								
Serum sodium levels								
≤145 mmol/L	Ref		Ref		Ref		Ref	
>145 mmol/L	1.67 (0.98–2.85)	0.059	2.34 (1.36–4.02)	0.002	2.13 (1.21–3.74)	0.009	2.22 (1.21–4.08)	0.010
Serum sodium classes								
Class 1	Ref		Ref		Ref		Ref	
Class 2	2.17 (1.52–3.08)	<0.001	2.37 (1.67–3.38)	<0.001	2.27 (1.59–3.25)	<0.001	2.31 (1.60–3.33)	<0.001
Class 3	1.72 (0.55–5.45)	0.355	2.47 (0.78–7.85)	0.124	2.48 (0.77–7.98)	0.129	2.95 (0.90–9.66)	0.074
Class 4	1.46 (0.81–2.61)	0.208	1.26 (0.70–2.27)	0.434	1.10 (0.61–1.99)	0.762	1.16 (0.63–2.14)	0.628

Model 1, no adjustment; Model 2, adjusted for age and gender; Model 3, Model 2 plus GCS, APSIII, OASIS, SOFA, SAPSII, CCI; Model 4, Model 3 plus heart failure, diabetes, CKD, vasopressor use, temperature, calcium, serum creatinine, hemoglobin, RBC, BUN.

### Subgroup analysis and sensitivity test

Subgroup analyses (Table 4) based on age, sex, CKD, diabetes, heart failure, and vasopressor use showed that the correlation between serum sodium classes and 30-day mortality was consistent in the remaining groups of patients with TBI with different characteristics, except for a certain interaction between age and serum sodium trajectory (*p* = 0.045). Sensitivity analyses revealed that the results were robust when based on interpolated data (Supplementary Table 5). Additionally, excluding Class 3 did not alter the association patterns between the remaining categories and 30-day and 90-day mortality (Supplementary Table 6).

**Table 4:** Subgroup analysis of serum sodium classes and 30-day mortality.

Subgroups	Class 1	Class 2	Class 3	Class 4	*P* for interaction
Age					0.045
>60	Ref	4.26 (2.12–8.57)	2.55 (0.33–19.37)	3.65 (1.2–11.09)	
≤60	Ref	1.56 (0.99–2.45)	2.03 (0.5–8.28)	0.89 (0.43–1.86)	
Gender					0.846
Female	Ref	1.6 (0.91–2.82)	1.84 (0.25–13.42)	1.17 (0.46–2.97)	
Male	Ref	2.23 (1.38–3.59)	1.85 (0.45–7.62)	1.49 (0.67–3.3)	
Vasopressor use					0.204
No	Ref	1.79 (1.01–3.15)	2.05 (0.49–8.47)	0.61 (0.19–1.98)	
Yes	Ref	2.08 (1.29–3.37)	1.42 (0.2–10.33)	2.44 (1.19–5.01)	
Heart failure					0.543
No	Ref	2.23 (1.49–3.32)	1.47 (0.36–6.02)	1.46 (0.72–2.93)	
Yes	Ref	1.31 (0.49–3.48)	5.23 (0.69–39.85)	0.9 (0.27–3.03)	
CKD					0.501
No	Ref	1.82 (1.22–2.71)	1.45 (0.36–5.91)	0.99 (0.46–2.16)	
Yes	Ref	3.88 (1.52–9.87)	2.46 (0.31–19.24)	2.46 (0.84–7.21)	
Diabetes					0.946
No	Ref	2.14 (1.38–3.34)	1.73 (0.42–7.09)	1.44 (0.68–3.03)	
Yes	Ref	1.76 (0.92–3.38)	2.17 (0.3–15.95)	1.23 (0.43–3.49)	

## Discussion

Most previous studies have focused on the prognostic value of static serum sodium indicators in TBI patients, but the fact remains that patients’ serum sodium levels change dynamically with time and treatment. This research sought to analyze the link between serum sodium levels and the early prognosis of TBI in ICU patients, which facilitated earlier detection of mortality risk and a better understanding of disease severity in TBI patients, thereby guiding clinical practice and improving patient outcomes.

Sodium is the primary determinant of plasma osmotic pressure, and when the human body is in a diseased state, it can affect the regulatory mechanisms of water and sodium metabolism, disrupting fluid homeostasis (16). Serum sodium disorder, including hyponatremia and hypernatremia, is a common electrolyte disorder in clinical practice. Both hyponatremia and hypernatremia are related to the increase of incidence rate and mortality (17–19). The study explored the influence of starting serum sodium levels and their progression on the likelihood of 30-day and 90-day mortality among TBI patients in the ICU. Our results showed that high serum sodium levels during the first ICU admission increased the risk of death at 30 and 90 days, patients with TBI in Class 2 had an increased risk of 30- and 90-day mortality compared with Class 1, Class 1 is manifested by an initial serum sodium level of around 140 mmol/L, which remains stable. Class 2 was characterized by an initial serum sodium level of 130-135 mmol/L, followed by a gradual increase in serum sodium levels. No difference in mortality risk was found between patients in Class 3 and Class 4 compared to those in Class 1. The initial serum sodium level in Class 3 was around 125–130 mmol/L, lower than in Class 2, and rises rapidly to a position of about 145 mmol/L. Although Class 3 rose faster than Class 2, it then rapidly decreases to normal levels (140–145 mmol/L); Class 4 showed that although the initial serum sodium level is high (140–145 mmol/L), which first decreased and then gradually increased to around 140 mmol/L. Both Class 3 and Class 4 patients showed a decrease in serum sodium levels, which indicated that the intervention measures reduced the mortality rate of TBI patients by lowering their serum sodium levels.

Our study indicated that Class 2 was associated with increased 30-day and 90-day mortality rates, but the underlying clinical mechanisms remained unclear. In response to this finding, we proposed the following hypothesis: this unique serum sodium change trajectory might reflect subacute sodium homeostasis dysfunction caused by hypothalamic–pituitary axis dysfunction following TBI, with potential mechanisms including the persistent presence of antidiuretic hormone deficiency (AVP-D). From a clinical perspective, this finding suggested that patients exhibiting this Class 2 pattern might require the increased frequency of dynamic monitoring of serum sodium levels and early initiation of targeted intervention measures, such as strict fluid management and electrolyte regulation. A recently published study outlined the acute-phase treatment for AVP-D as follows (20). An initial subcutaneous injection of desmopressin (1–2 μg) was administered, with dose adjustments made based on urine output. For fluid management, awake patients were allowed to drink fluids freely, while comatose patients required intravenous fluid replacement. Monitoring parameters included strictly recording fluid intake and output, monitoring changes in serum sodium levels, and avoiding overcorrection. For patients with persistent AVP-D, oral desmopressin should be used, with the dosage adjusted according to the patient’s condition. Prior to discharge, the recovery of endogenous AVP secretion should be assessed. During the recovery period, the need for long-term treatment could be confirmed through a water deprivation test or hypertonic saline stimulation test.

Research has shown that the occurrence of hypernatremia in individuals with brain injuries surpasses 35% (9, 21, 22). Hypernatremia was a significant predictor of mortality in patients admitted to neurological intensive care units (23–25), and its persistent presence was linked to higher mortality rates, prolonged hospital stays, and post discharge mortality rate in critically ill patients (26). The reasons for poor prognosis in TBI patients caused by hypernatremia included as follows. Hypernatremia could reduce left ventricular systolic force and impair cardiac function (27, 28). Moreover, it could lead to myelinolysis and neuronal necrosis, causing secondary brain injury (10, 29). Additionally, the osmotic gradient of hypernatremia can cause water to flow out of cells, leading to cell dehydration. Mild cases manifested as weakness, drowsiness, and fatigue, while in severe cases, a series of neurological manifestations, such as seizures, could occur. If the cerebral vein tears, intracranial and subarachnoid hemorrhage could also occur (30, 31). Neurological damage could also lead to prolonged mechanical ventilation, endangering the prognosis of patients (32). Hypernatremia was commonly linked to various neuromuscular symptoms, including muscle weakness and spasms (33). In addition, hypernatremia exacerbated insulin resistance. They could increase angiotensin II activity, reducing microvascular responsiveness to insulin, and leading to insulin resistance and hyperglycemia (34). In turn, Hyperglycemia induced increased insulin secretion, establishing a vicious cycle that could exacerbate or precipitate type 2 diabetes. This condition was linked to an increased frequency of multiple complications, ultimately affecting patient prognosis (35). However, it is still unclear how serum sodium levels affect the risk of mortality in patients suffering from TBI.

This study utilized data from multiple serum sodium measurements to construct a trajectory of serum sodium levels during the early phase of ICU admission in patients with TBI. Our goal was to examine the prognostic impact of both the serum sodium trajectory (longitudinal trajectory analysis) and the initial serum sodium levels (cross-sectional analysis). It helped to detect the risk of death in TBI patients at an early stage and also helped physicians to make timely and effective clinical decisions. However, it is undeniable that this study has certain limitations. Firstly, the data in this study were derived from a single center. Given the potential for significant variations in ICU treatment protocols, patient management practices, and the demographic characteristics of patients admitted to different healthcare institutions, the applicability of our findings to other settings could be limited. Future multicenter prospective studies were warranted to validate these findings. Secondly, we analyzed the effect of serum sodium trajectory within 96 h of admission to the ICU on the mortality at 30 days and 90 days, and the effect on the longer follow-up time (1 year, 3 years) needs more analysis. Third, although this study adjusted for some confounders, it still could not exclude the possibility that there might be some potential confounders that bias the results. For example, neuroimaging findings, dynamic changes in the GCS, and intracranial pressure management strategies were critical factors in the prognosis of TBI and could interact with serum sodium levels. The absence of these factors impaired the model’s ability to comprehensively assess prognostic factors. Future studies should integrate neuroimaging data and refined metrics of neurocritical care to enhance analysis. Additionally, the small sample size of some serum sodium trajectory subgroups could reduce the statistical power to detect differences between groups and affect the reliability of the conclusions. Thus, validation of our findings in larger cohorts was necessary. Lastly, this study included data analyzed from patients admitted to the ICU, which could not be applicable to non-ICU patients with TBI.

## Conclusion

After adjusting for confounding factors, elevated initial serum sodium (>145 mmol/L) was connected to a higher likelihood of death within 30 and 90 days for those with TBI. Among the different trajectories of serum sodium fluctuations, Class 2 (slow-growth type) was linked to a higher risk of mortality at both 30 and 90 days, whereas the other trajectories showed no such association. Future research should focus on elucidating the role of serum sodium levels and their trajectories in predicting mortality in TBI patients.

## Data Availability

Publicly available datasets were analyzed in this study. This data can be found at: https://mimic.mit.edu/docs/iv/.
