# Supplementary material for: Impact of serum sodium trajectory on 30-day mortality in traumatic brain injury patients: insights from a retrospective cohort study using MIMIC-IV database
Source: Front Neurol. 2025 Jul 25;16:1618586. doi: 10.3389/fneur.2025.1618586 (PMC12331721; doi:10.3389/fneur.2025.1618586)
Supplement: Supplementary file 2 [file Table_2.DOCX]

**Table 1.** Statistics for choosing the best number of classes

| **Class** | **Log likelihood** | **AIC** | **BIC** | **Entropy** | **%class1** | **%class2** | **%class3** | **%class4** | **%class5** | **%class6** |
| --- | --- | --- | --- | --- | --- | --- | --- | --- | --- | --- |
| 1 | -9061.66 | 18137.32 | 18170.55 | 1.0000 | 1.00 |  |  |  |  |  |
| 2 | -8876.91 | 17777.83 | 17834.80 | 0.6710 | 79.93 | 20.07 |  |  |  |  |
| 3 | -8824.81 | 17683.61 | 17764.32 | 0.6874 | 73.12 | 19.72 | 7.16 |  |  |  |
| 4 | -8803.33 | 17650.67 | 17755.11 | 0.7142 | 70.77 | 20.31 | 1.53 | 7.39 |  |  |
| 5 | -8788.62 | 17631.25 | 17759.43 | 0.7657 | 18.08 | 71.60 | 1.06 | 1.76 | 7.51 |  |
| 6 | -8796.45 | 17656.90 | 17808.82 | 0.5999 | 19.13 | 6.34 | 22.77 | 40.02 | 4.11 | 7.63 |

**Table 2** Univariate Cox regression models were used to screen for confounders associated with 30-day mortality in patients with TBI.

| Variables | HR (95% CI) | P |
| --- | --- | --- |
| Age | 1.03(1.02-1.04) | <0.001 |
| Gender(%) |  |  |
| Female | Ref |  |
| Male | 0.62(0.45-0.87) | 0.005 |
| Race (%) |  |  |
| White | Ref |  |
| Black | 0.91(0.42-1.97) | 0.803 |
| Others | 1.10(0.79-1.55) | 0.576 |
| SOFA | 1.11(1.06-1.16) | <0.001 |
| APSIII | 1.02(1.01-1.03) | <0.001 |
| GCS | 0.95(0.90-0.995) | 0.033 |
| CCI | 1.23(1.17-1.29) | <0.001 |
| SAPSII | 1.04(1.03-1.06) | <0.001 |
| OASIS | 1.05(1.03-1.08) | <0.001 |
| Temperture,℉ | 0.95(0.93-0.98) | 0.001 |
| Heart rate,bpm | 1.00(0.99-1.01) | 0.967 |
| Respiratory rate,insp/min | 0.99(0.95-1.03) | 0.593 |
| SBP,mmHg | 1.00(0.99-1.01) | 0.974 |
| DBP, mmHg | 0.99(0.98-1.00) | 0.143 |
| SpO2,% | 0.98(0.94-1.03) | 0.397 |
| Hemoglobin,g/dl | 0.87(0.81-0.95) | 0.001 |
| RBC,m/ul | 0.64(0.51-0.81) | <0.001 |
| Calcium,mg/dl | 1.26(1.04-1.53) | 0.021 |
| Potassium,mEq/L | 1.15(0.90-1.48) | 0.252 |
| Creatinine, mg/dl | 2.00(1.14-3.51) | 0.016 |
| Glucose,mg/dl | 1.005(1.001-1.01) | 0.012 |
| Platelet, k/ul | 0.998(0.996-1.001) | 0.177 |
| WBC, k/ul | 1.00(0.97-1.04) | 0.935 |
| BUN, mg/dl | 1.06(1.03-1.08) | <0.001 |
| PT,s | 1.01(0.99-1.03) | 0.22 |
| PTT,s | 1.01(1.00-1.02) | 0.092 |
| Ventilation ues (%) |  |  |
| No | Ref |  |
| Yes | 1.88(0.92-3.84) | 0.082 |
| Vasopressor use (%) |  |  |
| No | Ref |  |
| Yes | 1.49(1.07-2.07) | 0.019 |
| Diuretic use (%) |  |  |
| No | Ref |  |
| Yes | 1.06（0.75-1.50） | 0.744 |
| Variables | HR (95% CI) | P |
| Hypertension(%) |  |  |
| No | Ref |  |
| Yes | 1.15（0.83-1.60） | 0.405 |
| CKD (%) |  |  |
| No | Ref |  |
| Yes | 2.32（1.49-3.59） | <0.001 |
| Diabetes(%) |  |  |
| No | Ref |  |
| Yes | 2.08(1.47-2.94） | <0.001 |
| Heart failure(%) |  |  |
| No | Ref |  |
| Yes | 2.12（1.41-3.19） | <0.001 |
| Myocardial infarction(%) |  |  |
| No | Ref |  |
| Yes | 0.29（0.04-2.06） | 0.214 |
| COPD(%) |  |  |
| No | Ref |  |
| Yes | 1.34(0.74-2.41) | 0.338 |

**Table 3** Proportion of missing variables

| Variables | Missing(%) |
| --- | --- |
| GCS | 0.2 |
| SBP | 0.6 |
| DBP | 0.6 |
| Temperture | 0.2 |
| Hemoglobin | 0.6 |
| RBC | 0.5 |
| WBC | 0.5 |
| PT | 4.2 |
| PTT | 4.6 |
| Platelet | 0.7 |
| BUN | 0.1 |

**Table 4** Baseline characteristics of TBI patients by 4 serum sodium classes

| Variable | Overall | Class 1 | Class 2 | Class 3 | Class 4 | *p* |
| --- | --- | --- | --- | --- | --- | --- |
| N | 852 | 603 | 173 | 13 | 63 |  |
| Age | 64.00 (44.00 - 78.00) | 64.00 (46.00 - 79.00) | 60.00 (35.00 - 74.00) | 52.00 (30.00 - 64.00) | 71.00 (56.00 - 81.00) | 0.007 |
| Gender (%) |  |  |  |  |  | 0.564 |
| Female | 275(32.28) | 188 (31.18) | 62(35.84) | 3 (23.08) | 22 (34.92) |  |
| Male | 577(67.72) | 415(68.82) | 111(64.16) | 10 (76.92) | 41(65.08) |  |
| Race (%) |  |  |  |  |  | 0.548 |
| White | 460(53.99) | 335(55.56) | 83 (47.98) | 6 (46.15) | 36(57.14) |  |
| Black | 47 (5.52) | 34(5.64) | 8 (4.62) | 1(7.69) | 4(6.35) |  |
| Others | 345 (40.49) | 234(38.81) | 82 (47.40) | 6 (46.15) | 23 (36.51) |  |
| SOFA | 4.00 (2.00 - 6.00) | 4.00 (2.00 - 5.00) | 4.00 (2.00 - 6.00) | 4.00 (3.00 - 5.00) | 3.00 (2.00 - 7.00) | 0.687 |
| APSIII | 39.00 (29.00 - 49.00) | 38.00 (29.00 - 49.00) | 39.00 (30.00 - 52.00) | 37.00 (30.00 - 41.00) | 40.00 (29.00 - 55.00) | 0.429 |
| GCS | 15.00 (12.00 - 15.00) | 15.00 (12.00 - 15.00) | 15.00 (12.00 - 15.00) | 15.00 (15.00 - 15.00) | 14.00 (12.00 - 15.00) | 0.022 |
| CCI | 3.00 (1.00 - 5.00) | 3.00 (1.00 - 5.00) | 3.00 (1.00 - 5.00) | 3.00 (0.00 - 6.00) | 4.00 (1.00 - 6.00) | 0.175 |
| SAPSII | 34.16 ± 11.59 | 34.05 ± 11.75 | 34.34 ± 11.30 | 32.46 ± 9.61 | 35.05 ± 11.34 | 0.816 |
| OASIS | 33.35 ± 7.24 | 33.43 ± 7.15 | 33.38 ± 7.42 | 30.08 ± 7.01 | 33.17 ± 7.63 | 0.397 |
| Temperture, ℉ | 98.40 (97.70 - 99.10) | 98.30 (97.70 - 99.10) | 98.50 (97.70 - 99.20) | 98.90 (98.30 - 99.30) | 98.50 (97.80 - 99.10) | 0.231 |
| Heart rate, bpm | 87.30 ± 19.06 | 87.02 ± 18.78 | 86.64 ± 18.60 | 95.31 ± 17.57 | 90.06 ± 22.83 | 0.345 |
| Respiratory rate,insp/min | 18.54 ± 4.42 | 18.42 ± 4.45 | 18.74 ± 4.18 | 20.15 ± 4.79 | 18.76 ± 4.66 | 0.470 |
| SBP,mmHg | 128.65 ± 23.43 | 128.93 ± 23.32 | 127.45 ± 24.50 | 126.69 ± 15.70 | 129.59 ± 23.18 | 0.529 |
| DBP, mmHg | 72.02 ± 16.13 | 72.47 ± 16.15 | 70.02 ± 15.40 | 78.92 ± 17.16 | 71.65 ± 17.30 | 0.206 |
| SpO2, % | 100 (97 - 100) | 100 (97 - 100) | 100 (98 - 100) | 100 (97 - 100) | 99 (96 - 100) | 0.002 |
| Hemoglobin, g/dl | 11.37 ± 2.06 | 11.42 ± 2.05 | 11.43 ± 2.09 | 11.04 ± 2.27 | 10.81 ± 1.94 | 0.183 |
| RBC, m/ul | 3.72 ± 0.69 | 3.74 ± 0.68 | 3.73 ± 0.72 | 3.63 ± 0.68 | 3.53 ± 0.70 | 0.193 |
| Calcium, mg/dl | 8.29 ± 0.85 | 8.28 ± 0.81 | 8.27 ± 0.87 | 8.20 ± 1.07 | 8.45 ± 1.12 | 0.806 |
| Potassium, mEq/L | 4.07 ± 0.65 | 4.09 ± 0.64 | 4.01 ± 0.72 | 4.21 ± 0.86 | 4.11 ± 0.59 | 0.166 |
| Creatinine, mg/dl | 0.89 ± 0.27 | 0.88 ± 0.27 | 0.90 ± 0.27 | 0.86 ± 0.27 | 0.91 ± 0.29 | 0.821 |
| Glucose, mg/dl | 137.57 ± 37.10 | 136.45 ± 36.53 | 144.28 ± 38.43 | 131.05 ± 35.69 | 131.23 ± 37.44 | 0.029 |
| Platelet, k/ul | 191.29 ± 70.55 | 189.08 ± 68.79 | 197.57 ± 74.36 | 215.15 ± 79.03 | 190.24 ± 74.44 | 0.445 |
| WBC, k/ul | 11.67 ± 4.44 | 11.47 ± 4.32 | 12.75 ± 4.51 | 10.75 ± 3.73 | 10.87 ± 5.06 | 0.001 |
| BUN, mg/dl | 15.24 ± 6.07 | 15.38 ± 5.97 | 14.77 ± 5.84 | 12.43 ± 4.46 | 15.73 ± 7.62 | 0.330 |
| PT, s | 12.90 (11.90 - 14.25) | 12.90 (11.90 - 14.30) | 13.00 (11.90 - 14.10) | 12.20 (11.20 - 12.80) | 13.10 (11.90 - 14.20) | 0.246 |
| PTT,s | 27.80 (25.40 - 30.50) | 27.90 (25.50 - 30.50) | 27.10 (24.90 - 30.10) | 28.10 (25.60 - 30.30) | 28.90 (25.40 - 31.10) | 0.164 |
| Ventilation use (%) |  |  |  |  |  | 0.510 |
| No | 84 (9.86) | 65 (10.78) | 12 (6.94) | 1 (7.69) | 6 (9.52) |  |
| Yes | 768(90.14) | 538 (89.22) | 161(93.06) | 12 (92.31) | 57 (90.48) |  |
| Vasopressor use (%) |  |  |  |  |  | 0.263 |
| No | 451(52.93) | 323 (53.57) | 82(47.40) | 8(61.54) | 38(60.32) |  |
| Yes | 401(47.07) | 280 (46.43) | 91(52.60) | 5(38.46) | 25(39.68) |  |
| Diuretic use (%) |  |  |  |  |  | <0.001 |
| No | 574(67.37) | 428(70.98) | 92(53.18) | 7 (53.85) | 47 (74.60) |  |
| Yes | 278(32.63) | 175 (29.02) | 81(46.82) | 6 (46.15) | 16 (25.40) |  |
| Hypertension (%) |  |  |  |  |  | 0.743 |
| No | 496(58.22) | 354(58.71) | 96 (55.49) | 9 (69.23) | 37(58.73) |  |
| Yes | 356 (41.78) | 249 (41.29) | 77 (44.51) | 4 (30.77) | 26(41.27) |  |
| CKD (%) |  |  |  |  |  | 0.049 |
| No | 779 (91.43) | 557 (92.37) | 159 (91.91) | 11(84.62) | 52(82.54) |  |
| Yes | 73 (8.57) | 46 (7.63) | 14 (8.09) | 2 (15.38) | 11 (17.46) |  |
| Diabetes (%) |  |  |  |  |  | 0.887 |
| No | 676(79.34) | 476(78.94) | 140(80.92) | 11 (84.62) | 49 (77.78) |  |
| Yes | 176 (20.66) | 127 (21.06) | 33 (19.08) | 2(15.38) | 14 (22.22) |  |
| Heart failure (%) |  |  |  |  |  | 0.288 |
| No | 756(88.73) | 534 (88.56) | 158(91.33) | 12(92.31) | 52(82.54) |  |
| Yes | 96(11.27) | 69(11.44) | 15 (8.67) | 1 (7.69) | 11 (17.46) |  |
| Myocardial infarction (%) |  |  |  |  |  | 0.902 |
| No | 833(97.77) | 590 (97.84) | 169 (97.69) | 13 (100.00) | 61(96.83) |  |
| Yes | 19 (2.23) | 13(2.16) | 4. (2.31) | 0 (0.00) | 2 (3.17) |  |
| COPD (%) |  |  |  |  |  | 0.704 |
| No | 796(93.43) | 560(92.87) | 165 (95.38) | 12(92.31) | 59(93.65) |  |
| Yes | 56 (6.57) | 43 (7.13) | 8 (4.62) | 1 (7.69) | 4 (6.35) |  |
| 30-day mortality (%) |  |  |  |  |  | 0.003 |
| Alive | 710 (83.33) | 520 (86.24) | 129(74.57) | 10 (76.92) | 51 (80.95) |  |
| Dead | 142(16.67) | 83(13.76) | 44(25.43) | 3(23.08) | 12(19.05) |  |
| 90-day mortality (%) |  |  |  |  |  | <0.001 |
| Alive | 702 (82.39) | 518 (85.90) | 124 (71.68) | 10 (76.92) | 50(79.37) |  |
| Dead | 150(17.61) | 85(14.10) | 49 (28.32) | 3 (23.08) | 13(20.63) |  |

**Table 5** Sensitivity analysis results

|  | Model 1 | | Model 2 | | Model 3 | | Model 4 | |
| --- | --- | --- | --- | --- | --- | --- | --- | --- |
|  | HR | *P* | HR | *P* | HR | *P* | HR | *P* |
| **30-day mortality** |  | | | | | | | |
| serum sodium levels |  |  |  |  |  |  |  |  |
| ≤145mmol/L | Ref |  | Ref |  | Ref |  | Ref |  |
| ＞145mmol/L | 1.64(0.95-2.85) | 0.079 | 2.29(1.31-4.01) | 0.004 | 2.06(1.15-3.70) | 0.015 | 2.06(1.09-3.89) | 0.025 |
| serum sodium classes |  |  |  |  |  |  |  |  |
| Class 1 | Ref |  | Ref |  | Ref |  | Ref |  |
| Class 2 | 1.98(1.38-2.86) | <0.001 | 2.17(1.50-3.14) | <0.001 | 2.06(1.42-2.99) | <0.001 | 2.06(1.41-2.99) | <0.001 |
| Class 3 | 1.76(0.56-5.56) | 0.337 | 2.50(0.79-7.93) | 0.120 | 2.47(0.77-7.98) | 0.13 | 2.76(0.84-9.05) | 0.093 |
| Class 4 | 1.37(0.75-2.51) | 0.309 | 1.19(0.65-2.19) | 0.571 | 1.02(0.55-1.90) | 0.94 | 1.05(0.56-1.98) | 0.871 |
| **90-day mortality** |  |  |  |  |  |  |  |  |
| serum sodium levels |  |  |  |  |  |  |  |  |
| ≤145mmol/L | Ref |  | Ref |  | Ref |  | Ref |  |
| ＞145mmol/L | 1.67(0.98-2.85) | 0.059 | 2.34(1.36-4.02) | 0.002 | 2.13(1.21-3.74) | 0.009 | 2.14(1.17-3.94) | 0.014 |
| serum sodium classes |  |  |  |  |  |  |  |  |
| Class 1 | Ref |  | Ref |  | Ref |  | Ref |  |
| Class 2 | 2.17(1.52-3.08) | <0.001 | 2.37(1.67-3.38) | <0.001 | 2.27(1.59-3.25) | <0.001 | 2.27(1.58-3.26) | <0.001 |
| Class 3 | 1.72(0.55-5.45) | 0.355 | 2.47(0.78-7.85) | 0.124 | 2.48(0.77-8.00) | 0.128 | 2.84(0.87-9.28) | 0.085 |
| Class 4 | 1.46(0.81-2.61） | 0.208 | 1.26(0.70-2.27) | 0.434 | 1.10(0.61-1.99) | 0.76 | 1.14(0.62-2.09) | 0.671 |

Model 1, no adjustment; Model 2, adjusted for age and gender; Model 3,Model 2 plus GCS, APSIII, OASIS, SOFA, SAPSII, CCI; Model 4, Model 3 plus heart failure, diabetes, CKD, vasopressor use, temperature, calcium, serum creatinine, hemoglobin, RBC, BUN.

**Table 6** Sensitivity analysis results

|  | Model 1 | | Model 2 | | Model 3 | | Model 4 | |
| --- | --- | --- | --- | --- | --- | --- | --- | --- |
|  | HR | *P* | HR | *P* | HR | *P* | HR | *P* |
| **30-day mortality** |  | | | | | | | |
| serum sodium classes |  |  |  |  |  |  |  |  |
| Class 1 | Ref |  | Ref |  | Ref |  | Ref |  |
| Class 2 | 1.98(1.37-2.86) | <0.001 | 2.17(1.50-3.13) | <0.001 | 2.06(1.42-2.99) | <0.001 | 2.10(1.44-3.08) | <0.001 |
| Class 4 | 1.37(0.75-2.51) | 0.309 | 1.19(0.65-2.19) | 0.569 | 1.02(0.55-1.88) | 0.959 | 1.06(0.57-2.00) | 0.848 |
| **90-day mortality** |  |  |  |  |  |  |  |  |
| serum sodium classes |  |  |  |  |  |  |  |  |
| Class 1 | Ref |  | Ref |  | Ref |  | Ref |  |
| Class 2 | 2.17(1.52-3.08) | <0.001 | 2.37(1.67-3.38) | <0.001 | 2.27(1.59-3.25) | <0.001 | 2.33(1.62-3.36) | <0.001 |
| Class 4 | 1.46(0.81-2.61） | 0.208 | 1.26(0.71-2.27) | 0.432 | 1.09(0.60-1.97) | 0.778 | 1.15(0.63-2.11) | 0.651 |

Model 1, no adjustment; Model 2, adjusted for age and gender; Model 3,Model 2 plus GCS, APSIII, OASIS, SOFA, SAPSII, CCI; Model 4, Model 3 plus heart failure, diabetes, CKD, vasopressor use, temperature, calcium, serum creatinine, hemoglobin, RBC, BUN.
